# Next generation Europe: a recovery plan for children, adolescents and their families: For the time after the pandemic, we need a vision and investments for the future

**DOI:** 10.1007/s00787-021-01767-w

**Published:** 2021-04-10

**Authors:** Jörg M. Fegert, Laura A. Kehoe, Fusun Çuhadaroglu Çetin, Maeve Doyle, Stephan Eliez, Johannes Hebebrand, Manon Hillegers, Andreas Karwautz, Konstantinos Kotsis, Eniko Kiss, Milica Pejovic‐Milovancevic, Anne-Marie Räberg Christensen, Jean-Philippe Raynaud, Dimitris Anagnostopoulos

**Affiliations:** 1 Department of Child and Adolescent Psychiatry, Psychosomatics and Psychotherapy, University of Ulm, Ulm, Germany; 2 Medical Communications, Avenue des Cadolles 12D, 2000 Neuchatel, Switzerland; 3 Hacettepe University School of Medicine, Ankara, Turkey; 4 Eastern Connecticut State University, Willimantic, USA; 5 Department of Psychiatry, Faculty of Medicine, Geneva University Hospitals, Geneva, Switzerland; 6 Department of Child and Adolescent Psychiatry, Psychosomatics and Psychotherapy, LVR Klinikum Essen, University Hospital Essen, University of Duisburg-Essen, Essen, Germany; 7 Child and Adolescent Psychiatry/Psychology, Erasmus Medical Center Rotterdam, Rotterdam, Netherlands; 8 University Department of Child and Adolescent Psychiatry, Medical University of Vienna, Vienna, Austria; 9 Psychiatry Department of Psychiatry Faculty of Medicine School of Health Sciences, University of Ioannina, Ioannina, Greece; 10 Eniko Kiss, Clinical Medicine, University of Szeged, Szeged, Hungary; 11 Department of Psychiatry, School of Medicine, University of Belgrade, Belgrade, Serbia; 12 Child and Adolescent Psychiatric Center, Glostrup, Denmark; 13 Service Universitaire de Psychiatrie de l’Enfant et de l’Adolescent (SUPEA), Universitaire de Toulouse, Toulouse, France; 14 Department of Child Psychiatry, Medical School, General Pediatric Hospital of Athens “Aghia Sophia”, National and Kapodistrian University of Athens, Athens, Greece

The youth of today—our most precious resource—are finally getting the attention they deserve. The impact of the COVID-19 pandemic, including the toll exerted on their mental health had been overlooked during the early months of the pandemic. In the first lockdown, the needs of children and adolescents and their families were largely ignored apart from the child and adolescent psychiatrists all over Europe who worked tirelessly on their behalf. The lives of our young people were severely restricted and for many, this complex situation was incomprehensible. The protection of these children’s rights and their welfare have finally come sharply into focus. But how can we ensure that this focus will continue? How can the neglect of children, adolescents and their families be remedied? What should the future hold for the next generation Europe after the acute phase of the pandemic?

The use of terms such as “lost generation” or “generation Corona” should be discouraged as this could foster a belief that they cannot be helped and that the neglect that they have suffered is irreparable. This position is neither legally, socially nor ethically justifiable. The constant calls by UNICEF and other organizations like ESCAP to respect children’s rights is not an aspirational one. These organizations ensure the fundamental rights of children to be heard and to have their situation considered. The pandemic is shedding light on this fact like never before. The protection of minors must therefore be articulated as a state objective with constitutional status.

## Thinking ahead

Child, youth and family policies must be forward thinking and must immediately set a clear and strong recovery plan for the present and the future. Two scenarios are predicted after successful management of the acute first wave of the pandemic. In one scenario, many children and their families will adapt and recover. In the other scenario, many are in danger of being left behind and suffering from the multiple consequences of the crisis for too long. We must anticipate and cater for the individual needs of both groups.

Now the EU has decided on a huge development program, "Next Generation EU" (https://ec.europa.eu/info/strategy/recovery-plan-europe_en). It is already clear that the psychological stress on children, adolescents and families during the pandemic has increased due to the lockdown and other pandemic control restrictions. Furthermore, children and adolescents who were mentally ill or had severe emotional problems before the crisis now have an increased need for therapy and support. ESCAP, therefore calls on the EU and the national governments in Europe not to abandon and neglect the situation of these children but to comprehensively include their needs in the plan. The backbone of our society is the nuclear family and the households in which we live. This structure has helped us continue to function as a society by maintaining education, employment, and the economy through homeschooling and working from home during the pandemic lockdowns. Some children and families have managed this situation well, but many have not. This change in our way of working and being educated has had major implications for the psychological well-being of parents and children. Supporting families and re-establishing contact and therapy with children, especially those children with pre-existing conditions, is therefore no longer only socially appropriate but economically meaningful and absolutely essential as an investment in the future.

## Reflecting on the past year, what can we learn?

One of the many adverse consequences of the COVID-19 pandemic with its lengthy lockdown phases has been the drastic reduction in the provision of child mental health, child welfare and child protection resources [1]. Clinics, youth welfare offices and independent organizations often recorded a deceptive decrease in contacts that did not correspond to the real needs of the population. In addition, for the best part of a year, children and adolescents experienced major reductions in social contacts normally available through school, social, leisure, sports or artistic activities, all of which are a source of validation and structuring [2]. The removal of these essential social outlets together with other members of the family trying to home school and work from home, combined with individuals with pre-existing conditions is a “perfect storm” for escalating tensions and conflicts within the home and increasing the risks of abuse [16].

Since the lockdown, child welfare risks are being reported much less frequently by those who are usually present in a child’s environment, such as teachers and caregivers. Youth welfare offices in Germany received 25% fewer hazard reports, and the German medical child protection [14] hotline registered a decrease in reports from practices and hospitals to 70% of the usual level [3, 17]. Worryingly, many schools reported that 20% of the children had virtually disappeared from the radar and were no longer available [4]. At the same time, there were more calls being made to helplines for children and young people but often without direct consequences meaning no active intervention or follow-up [5].

Following the first lockdown a “rebound effect” that is, an overshooting of the demands to far more than the usual number of consultations was observed. Studies and statistics emerged suggesting that psychological distress, poverty, aggression and violence are likely to have increased in many families [6–8]. One can predict that these problems will continue to worsen as more lockdowns are being implemented. Emotional and behavioural problems that previously affected just under a fifth of all children and adolescents now occur in just under a third [9]. Counselling centres, outpatient clinics and clinics report noticeable increases in anxiety disorders, depression, suicidality and eating disorders. School refusal is not systematically noticed at the moment, although there are several different causes in the context of the pandemic ranging from being afraid of an infection all the way to enjoying life without school. Therefore, it seems highly likely that the whole range of problems will emerge when everyday life gradually normalizes. If this is the case, society must be prepared for this phase.

These worrying statistics and trends need to trigger proactive action not merely increasing levels of concern.

What is clear, is that the situation across Europe for these children is extremely volatile and to stop this escalating further we need to act now and put strategic planning in place early.

## Impact of school closures

School closures have sparked huge concern and debate since the pandemic started. In some countries, politicians are emphasizing the importance of opening kindergartens and elementary schools as the health burdens for children and families are becoming more apparent [10]. The commitment of teachers has been exemplary, but students cannot be expected to be at the same academic level as in previous years. According to the United Nations (2021), 214 million children, which is equal to 1 in 7 children, missed more than three-quarters of their face-to-face learning, while over 888 million face disruption in their education due to full and partial closures [11]. Failure to take this reality into account, by maintaining end-of-year exams comparable to previous years, will lead to school and professional training disruptions that will be a source of marginalization and subsequent social precariousness. Conversely, by maintaining constant success rates, comparable to the support and loans conceded to economy, the resilience of pupils and students will be promoted over several years. It is therefore essential to permit primary and secondary school students, vocational training students and students in general to demonstrate their resilience by catching up with their studies over the next 2–3 years by keeping fixed failure and success rates based on the rates of previous years.

Furthermore, despite all the efforts of teachers and the advances in virtual learning techniques, many students will not succeed in achieving the same mastery of skills and academic achievements. With the anticipated increased need for help, rapid action will be necessary and this can only be achieved through sufficient financial resources within the educational system.

## What should child mental health experts be doing?

One of the central problems with a gradual reopening is a psychosocial one. If this goes unnoticed, the consequences for all of us will be enormous. As the pandemic wanes, the rapidly increasing need for help will become apparent in many families, especially in the case of children and adolescents who were impaired or at risk before the coronavirus crisis. It is astonishing at how rarely governments recognize what is absolutely foreseeable for us as medical experts. Hundreds of thousands of individuals will have to catch up on what they have missed: all the counselling, aid, self-help and therapy facilities, and of course, the support programs in schools will be challenged to the maximum.

Child and adolescent psychiatrists, psychotherapists and social services must have their proposals, plans and strategies ready. We strongly recommend that:

- Providing whole family strategies for coping during the pandemic. Social psychological observations and intervention research show that major crises can often have special effects due to pre-existing stress and trauma [12]. Thus, families that have already overcome a crisis, have learned constructive ways of dealing and working through the crisis together i.e., when they cook, learn, talk and play together. The aim is strengthening coping strategies for everyone.
- Working alongside the policy makers to make them fully aware of the needs and requirements of children during and after the immediate crisis.
- Additional personnel at offices and schools must be recruited and trained for individual support of children and families in need.
- Pre-existing files on specific children must be proactively searched and gaps recognized so that the necessary support can begin immediately, and those children that have gone missing in the system can be found and given the help and therapy they need.
- The long-term impact of a coronavirus infection is becoming more and more apparent in adults. With the new mutations the youngest age-group is now being infected more than other age-groups. Therefore, we need a surveillance system to track infection-related mental health issues, in addition, we need to train clinicians for the treatment of children suffering from the long-term consequences of the disease.
- Support for increasing capacity significantly in Child and Adolescent Mental Health Services immediately due to anticipated increase in referrals to the services.
- Personnel are already strained both physically and mentally, therefore, support networks for all systems and staff are needed to prevent information on youths being lost due to staff shortages and illness.

The ESCAP CovCAP survey launched in the early phase of the pandemic demonstrated that child and adolescent psychiatrists quickly adopted the use of video- or telemedicine and that many important relationships with children and adolescents could be maintained [13]. While this digitization is experiencing a significant boost during the crisis, there is a lack of youth-appropriate approaches to digital participation, and there is a lack of digitally supported acceleration in determining emotional and participation deficits. Until now, concepts that provide young people with more in-depth help using digital media are still lacking [15]. “How do I get legal, therapeutic or medical help?”, “Who can I turn to and what am I entitled to?” can be heard from these youths. It needs to become easier but safe for them to find answers on the internet and on social media. Thus, to maintain the additional benefits of this method of therapy and treatment, an investment in digitization and modernization of the administrative processes related to the provision of help and therapy is urgently required.

## Cost implications

Systems like counselling, aid, self-help and therapy were "shut down" in emergency mode due to the pandemic. Family helpers or self-help groups that had developed continuity and effectiveness over years had to pause because of the pandemic, impacting thousands of children and families. Governments naturally are giving considerable thought and attention to devising measures to help deal with the economic crisis. It is, however, quite clear that all economic crises have major effects on the mental health of our population.

Hence, a targeted investment program is needed here to prevent the gap between well-funded children and families and children in need of support drastically widening further in Europe and to address youth welfare and family policy. Efficient ring-fenced and targeted funding should be ensured to fully support these specific services. If this does not occur, it will not only be to the detriment of the vulnerable disadvantaged children and their families but will be to the detriment to society as a whole.

In practical terms, what is needed is a general, real-time update of the assistance planning via a real-time check-up. We have to actively determine where the affected children are now and work with new support in the summer months as soon as contact can resume.

The ultimate goal should be to strengthen the mental health system for children. Likewise, unaccompanied minors, asylum seekers and refugees, all of whom have also been negatively affected by the pandemic, should not be neglected and must be taken into consideration. It should be ensured that they receive the appropriate care within the National Health Systems and the child protection systems.

## Driving policy forward

Forward-looking policy focuses on the interests of future generations and is more important today than ever before. Fortunately, the attention of child protection and child welfare has now become a public issue.

At the start of the COVID-19 pandemic, no one could predict its course and it is still the case that we are none the wiser about when it will end. The imposition and lifting of lockdowns, the social restrictions, which are sometimes eased only then to be re-imposed, the excitement accompanying the development of effective vaccines only to be followed by very slow roll out of vaccination programmes with administrative difficulties at international, national and local levels has triggered anger and despair among the world’s citizens.

It is a fact that pandemics exacerbate inequalities and therefore, it is no surprise that child and adolescent mental health services, having been chronically underfunded, have been shown to be so deficient.

## Conclusions

All over Europe we urgently need a “Recovery Plan” for children. We need a well-thought-out psychosocial agenda to address and alleviate the pressures of children and families during the pandemic. Governments need to include all relevant stakeholders in devising this plan and streamlining administrative processes. The funding for this plan needs to be efficient, targeted, ring-fenced and of course adequate. The plan needs to be enacted quickly with an emphasis on repairing psychological impairment and preventing further manifestation of pathology, such as abuse, mental disorders, academic underachievement, poverty and unemployment.

Next Generation Europe—A Recovery Plan for Children, Adolescent and their Families’ needs to start now!
